# Hepatitis C Attributable Healthcare Costs and Mortality among Immigrants: A Population-Based Matched Cohort Study

**DOI:** 10.1155/2024/5573068

**Published:** 2024-02-24

**Authors:** Aysegul Erman, Yeva Sahakyan, Karl Everett, Christina Greenaway, Naveed Janjua, Jeffrey C. Kwong, William W. L. Wong, Hong Lu, Beate Sander

**Affiliations:** ^1^Toronto Health Economics and Technology Assessment Collaborative (THETA), University Health Network, Toronto, ON, Canada; ^2^Institute for Clinical Evaluative Sciences, Toronto, ON, Canada; ^3^Division of Infectious Diseases, Jewish General Hospital, McGill University, Montreal, QC, Canada; ^4^BC Centre for Disease Control, Vancouver, BC, Canada; ^5^University of Toronto, Toronto, ON, Canada; ^6^Public Health Ontario, Toronto, ON, Canada; ^7^School of Pharmacy, University of Waterloo, Kitchener, ON, Canada

## Abstract

**Background:**

Data on the economic burden of chronic hepatitis C (CHC) among immigrants are limited. Our objective was to estimate the CHC-attributable mortality and healthcare costs among immigrants in Ontario, Canada.

**Methods:**

We conducted a population-based matched cohort study among immigrants diagnosed with CHC between May 31, 2003, and December 31, 2018, using linked health administrative data. Immigrants with CHC (exposed) were matched 1 : 1 to immigrants without CHC (unexposed) using a combination of hard (index date, sex, and age) and propensity-score matching. Net costs (2020 Canadian dollars) collected from the healthcare payer perspective were calculated using a phase-of-care approach and used to estimate long-term costs adjusted for survival.

**Results:**

We matched 5,575 exposed individuals with unexposed controls, achieving a balanced match. The mean age was 47 years, and 52% was male. On average, 10.5% of exposed and 3.5% of unexposed individuals died 15 years postindex (relative risk = 2.9; 95% confidence interval (CI): 2.6–3.5). The net 30-day costs per person were $88 (95% CI: 55 to 122) for the prediagnosis, $324 (95% CI: 291 to 356) for the initial phase, $1,016 (95% CI: 900 to 1,132) for the late phase, and $975 (95% CI: −25 to 1,974) for the terminal phase. The mean net healthcare cost adjusted for survival at 15 years was $90,448.

**Conclusions:**

Compared to unexposed immigrants, immigrants infected with CHC have higher mortality rates and greater healthcare costs. These findings will support the planning of HCV elimination efforts among key risk groups in the province.

## 1. Introduction

Untreated chronic hepatitis C (CHC) can lead to advanced liver disease and is one of the major causes of liver cancer and liver-related death. Fortunately, highly effective direct-acting antivirals (DAAs) for the hepatitis C virus (HCV) offer the opportunity to eliminate HCV as a public health concern. The World Health Organization (WHO) has set a series of targets for HCV elimination by 2030 [1]. However, elimination will require considerable planning and investment in expanded screening and treatment initiatives [2].

Individuals living with CHC are highly heterogeneous. In Canada, a disproportionate number of CHC infections (∼30%) occur among foreign-born individuals [3]. Compared to nonimmigrants, immigrants can face long delays in diagnosis and can present with more advanced liver disease [4, 5]. Indeed, the health and economic consequences of delaying linkage to HCV treatment can be substantial, especially among those with serious liver disease [6, 7].

Reliable estimates of CHC-attributable healthcare costs are necessary for making appropriate resource allocation decisions among different populations and for healthcare planning to meet WHO targets. Several studies have examined CHC costs in Canada [8–10]; however, there are no data on the economic burden of CHC among immigrants. Our objective was to estimate CHC-attributable mortality and healthcare costs among immigrants in Ontario, using real-world population-level data.

## 2. Methods

### 2.1. Study Design, Setting, and Population

We conducted a population-based retrospective matched cohort study to estimate CHC-attributable healthcare costs among immigrants in the Canadian province of Ontario (population ∼14.9 million as of 2022) using an incidence-based costing approach. We included individuals with a record of CHC infection diagnosed between May 31, 2003, and December 31, 2018, among individuals who immigrated to Canada after 1985 with a valid Ontario health insurance plan (OHIP) number whose laboratory testing data could be linked to health administrative records held at ICES (formerly the Institute for Clinical Evaluative Sciences). We followed the study cohort up to March 31st, 2020, or until death. Consistent with the previously published study by our group [11], CHC was identified by a positive test result for HCV antibody or HCV ribonucleic acid (RNA) during the study timeframe, with the exclusion of cases with acute clearance of HCV defined by negative or undetectable HCV RNA test results within 12 months following the first positive HCV test with no record of Ontario Drug Benefit (ODB) claim for HCV antiviral treatment identified by drug identification number (DIN) listed in S.  Table 1. After excluding 158 individuals with missing data on age, sex, or socioeconomic covariates, and those aged >100 years, our study cohort included 6,914 individuals with CHC (hereafter referred to as “exposed”) who met eligibility. For the exposed individuals, the index date was the first date of HCV diagnosis.

Individuals without CHC (hereafter referred to as “unexposed”) were Ontario immigrants with no or negative HCV antibody or HCV RNA testing records who were alive at the start of the study period on May 31, 2003. We selected unexposed individuals by randomly sampling 5% of the Ontario population using the Registered Persons Database (RPDP), a population-based registry of all Ontario residents eligible for provincial health insurance. After excluding individuals with missing covariate data and those aged >100 years, 120,693 unexposed individuals who met the eligibility criteria were included. For unexposed individuals, we randomly assigned an index date based on the index date distribution of individuals with CHC, following their landing date in Ontario and between May 31, 2003, and December 31, 2018.

### 2.2. Data Sources

We obtained information on HCV exposure using HCV antibody and RNA testing records in the Public Health Ontario (PHO) laboratory dataset and linked them to health administrative, demographic, and immigration data held at the ICES to identify immigrants living with HCV and to collect demographic and healthcare utilization data. We accessed information on immigration status using the Immigration, Refugees, and Citizenship Canada (IRCC) Permanent Resident Database, which holds data on individuals who have been granted permanent resident status in Canada since 1985. We accessed demographic information on birth year, sex, rurality, and neighborhood income quintile from the RPDB. Markers of social marginalization, such as residential instability quintiles, material deprivation quintiles, and ethnic concentration quintiles, were obtained from Ontario's Marginalization Index Database (ONMARG). Information on human immunodeficiency virus (HIV) status, cirrhosis, decompensated cirrhosis (DC), hepatocellular carcinoma (HCC), liver transplant, death, substance use disorders related to drug and/or alcohol use, and comorbidities (Aggregated Diagnosis Groups (ADG) comorbidity classification scheme) were collected from the Canadian Institute for Health Information's Discharge Abstract Database (DAD) and National Ambulatory Care Reporting System (NACRS), OHIP, Ontario Mental Health Reporting System (OMHRS), Ontario Cancer Registry (OCR), and Office of the Registrar General Death registry (ORGD) datasets, using the diagnostic, procedure-related, and death codes listed in S.  Table 2-3. We used the Johns Hopkins Adjusted Clinical Group® (ACG®) system to determine the total number of ADGs over the year preceding the index date [12]. We compiled all baseline characteristics at the index date. All records were linked using unique identifiers and analyzed at ICES. Healthcare utilization and the corresponding costs were retrieved from the above-mentioned administrative databases and are described in more detail in the“Outcomes” section.

### 2.3. Phase-of-Care Approach

We used a phase-based approach to estimate CHC-attributable costs from diagnosis to death [8, 13, 14]. To do so, we allocated the total observation time of each individual to phases that represent the natural history of disease consistent with previous CHC costing studies [8]: (1) *prediagnosis phase,* defined as 6 months prior to index date, which capture costs associated with diagnostic testing and work-up to establish HCV diagnosis; (2) *initial care phase*, covers the time between CHC diagnosis up to start of either the late phase or the terminal phase or until the end of follow-up for those remaining alive throughout follow-up and without advanced liver disease; this phase captures the cost of active treatment or monitoring following initial diagnosis; (3) *late phase* starts at 3 months prior to the first diagnosis of advanced liver disease(decompensated cirrhosis, hepatocellular carcinoma, and liver transplantation) and captures ongoing surveillance for CHC-related complications and lasts up to the start of the terminal phase for those who died or up to end of follow-up for those remaining alive, and (4) *terminal phase,* representing the last 6 months prior to death for individuals who have died and captures the care received during the end-of-life period. The length of each phase was based on a previous study focused on CHC [8] and was validated by a clinical expert (CG). The starting points of each phase were indicative of clinically meaningful shifts in care patterns and costs.

### 2.4. Matched Cohort

We matched each immigrant with CHC to one immigrant without CHC using nearest-neighbor matching without replacement. Matching was performed using a combination of hard matching (sex, birth year, index year, presence of alcohol- or drug-related substance use disorder at index, and overall morbidity burden categories (within one year prior to index as measured by ADGs)) and propensity-score matching using a caliper width equal to 0.2 standard deviations of the logit of the propensity score [15]. The propensity algorithm included covariates collected at indexes such as age at diagnosis, neighborhood income quintile, residential instability quintile, material deprivation quintile, ethnic concentration quintile, dependency quintile, rurality, HIV coinfection, landing year, years of schooling at landing, and the total number of comorbidities within the one year prior to index diagnosis based on the total number of ADGs.

Exposed individuals were rematched to unexposed controls to estimate the phase-specific healthcare costs. For the late phase, an index date of three months prior to the date of advanced liver disease diagnosis was selected for exposed individuals, whereas for unexposed individuals, it was randomly assigned. The variables used for hard and propensity-score matching were the same as above. To estimate the terminal phase cost, exposed individuals who died during the study were rematched to unexposed individuals who also died during the study period. The index date was the date of death. Hard-match variables included index year, sex, and birth year. For unexposed individuals, the birth year was set to be earlier than or equal to that of the exposed individuals. The variables used for propensity-score matching were the same as described above, except for age at the time of diagnosis, which was replaced by age at death.

### 2.5. Outcomes

We estimated the cumulative incidence of all-cause mortality at 1 year, 5 years, 10 years, and 15 years postindex. Direct healthcare costs adjusted to 2020 Canadian dollars were estimated from the provincial public payer perspective. We used the ICES costing algorithm to obtain healthcare costs (standardized to 30 days)of the study cohort from the index date to the end of follow-up or death [16]. Phase-specific costs were calculated from the beginning of each phase to the start of the subsequent relevant phase or end of follow-up. Next, we combined phase-specific costs with survival data to estimate the long-term costs.

To explore the nature of resource use over time, costs were further stratified by healthcare service cost components as follows: (1) outpatient services (hospital outpatient clinics and visits to cancer clinics (NACRS)); (2) physician services (physician billings for inpatient and outpatient visits (OHIP)); (3) emergency department visits (NACRS); (4) same-day surgery (OHIP); (5) inpatient care (inpatient hospitalizations (DAD), admissions to designated mental health beds (OMHRS)); (6) outpatient prescription claims (ODB); (7) laboratory services (OHIP); and (8) other services (nonphysician services (OHIP), assistive devices program, rehabilitation (NRS), home care services (OACCAC HCD), complex continuing care (CCR), and long-term care).

### 2.6. Statistical Analyses

We assessed the quality of the match using standardized differences (SD), with SD less than 0.1 indicating negligible differences between covariates. We estimated CHC-attributable all-cause mortality by calculating the absolute risk reduction (ARR) and relative risks (RR) using conditional Poisson regression with robust variance estimators.

We calculated the attributable (net) costs by determining the mean difference between the total healthcare costs for the matched exposed and unexposed individuals using regression analysis. The 95% confidence intervals (CIs) were estimated using the standard errors of the regression coefficients. To estimate mean 1-year, 5-year, 10-year, and 15-year net costs, we combined phase-specific costs with crude survival data using the Yabroff equation (17). The margins of the 95% CIs of phase-specific costs were used to estimate the plausible range of mean net costs. Costs beyond one year were discounted by 1.5% annually [18]. Additionally, we stratified the results by age and sex. SAS Enterprise Guide 7.15 (SAS Institute, Inc.) was used for all statistical analyses.

## 3. Results

### 3.1. Study Cohort

Between May 2003 and December 2018, we identified 6,914 Ontario immigrants with CHC who met eligibility, and we were able to match 81% (*N* = 5,575) of immigrants with CHC to immigrants without CHC (Table 1). Compared to unexposed individuals, exposed individuals were older, had slightly earlier landing dates, were more likely to have markers of social marginalization (e.g., lower income and higher material deprivation levels), and were more likely to have drug- or alcohol-related substance use disorders and other comorbidities prior to matching. Following matching, we were able to achieve a balance in all characteristics with standardized differences of less than 0.1, except for the landing year (standardized difference: 0.38). On average, matched individuals were aged 46 years at the index date and had received approximately 11 years of education at the time of landing, 44% were baby boomers(born between 1945 and 1963), 52% were male, 99% lived in urban settings, ∼1% experienced drug- or alcohol-related substance use, <1% were infected with HIV, ∼35% experienced the highest levels of material deprivation, residential instability, and lowest income levels, and 59% experienced a low comorbidity burden (0–3 ADGs) 1 year prior to the index date. Unmatched immigrants with CHC were older at the time of diagnosis and had a higher comorbidity burden (>8 ADGs) and a higher proportion of drug- or alcohol-related substance use than matched individuals.

The demographic characteristics of the study cohort in the late and terminal phases, before and after matching, are shown in S.  Table 4 and S.  Table 5. In total, we identified 1,582 and 1,070 exposed individuals in the late and terminal phases, respectively. We were able to match 97% (*N* = 1,535) and ∼71% (*N* = 756) of the exposed individuals in the late and terminal phases, respectively, to achieve balanced matches.

### 3.2. Total and CHC-Attributable Mortality

On average, 1.3%, 4.5%, 8.4%, and 10.5% of exposed individuals and 0.2%, 1.2%, 2.6%, and 3.5% of unexposed individuals died at 1 year, 5 years, 10 years, and 15 years postindex, respectively (Table 2). Individuals with CHC were 6.7 (95% CI: 3.6–12.7) times more likely to die at 1 year, 3.9 (95% CI: 3.0–5.12) times more likely to die at 5 years, 3.3 (95% CI: 2.8–3.9) times more likely to die at 10 years, and 2.9 (95% CI: 2.6–3.5) times more likely to die at 15 years compared to unexposed individuals.

### 3.3. Total and CHC-Attributable Healthcare Costs by Phase of Care

Mean (median) lengths of initial and late phases were 8.5 (8.8) and 7.1 (6.7) years for matched exposed individuals and 7.4 (5.3) and 7.1 (7.7) years for unexposed individuals, respectively. The mean lengths of the prediagnostic and terminal phases were predefined, with a duration of 180 days each.

For the prediagnostic phase, the total 30-day healthcare costs were $247 per person for exposed and $159 per person for unexposed individuals, while CHC-attributable healthcare costs were $88 (95% CI: $55 to $122) (Table 3). In the prediagnostic phase, physician and laboratory services accounted for the main differences in health care costs for exposed compared to unexposed individuals. For the initial care phase, the total 30-day healthcare costs were $510 per person for exposed and $187 for unexposed individuals, translating to CHC-attributable costs of $324 (95% CI: $291 to $356) per person. In this phase, outpatient prescriptions accounted for important differences in costs between matched pairs. For the late phase, the total 30-day healthcare costs were $1,290 per person for exposed and $274 per person for unexposed individuals, translating to CHC-attributable costs of $1,016(95% CI: $901 to $1,132) per person. Differences in outpatient prescriptions followed by inpatient care and physician services accounted for the important differences in cost between matched pairs in this phase. For the terminal phase, 30-day healthcare costs were $8,967 per person for exposed and $7,992 for unexposed individuals, translating to CHC-attributable costs of $975 (95% CI: −25 to 1,974) per person, with main differences attributable to inpatient care.

**Figure 1:**
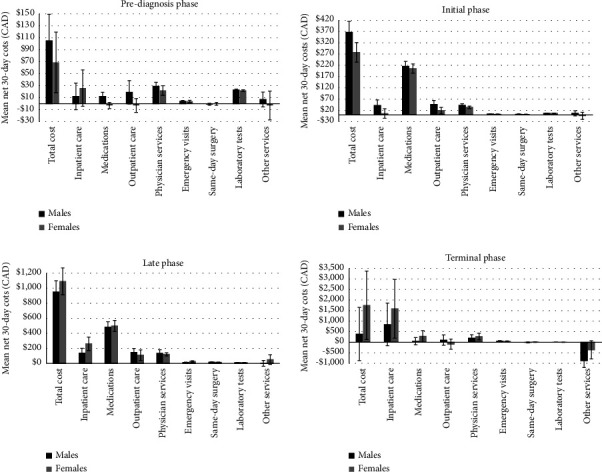
Net healthcare costs (in 2020 CAD) by phase of care for males and females. *Note*. Negative attributable cost observed, e.g., in the terminal phase for “other services,” often occurs because exposed individuals are hospitalized and do not incur costs outside the hospital, but unexposed do.

**Figure 2:**
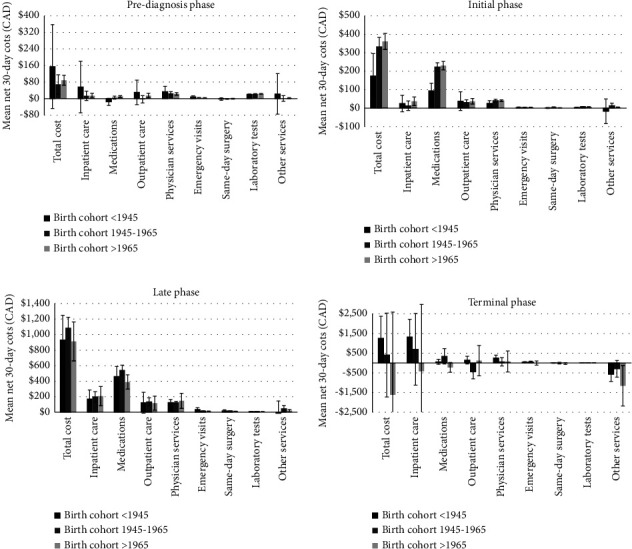
Net healthcare costs (in 2020 CAD) by phase of care by birth cohorts. *Note.* Negative attributable cost observed, e.g., in the terminal phase for “other services,” often occurs because exposed individuals are hospitalized and do not incur costs outside the hospital, but unexposed do.

Net 30-day costs were slightly lower for females than for males in the prediagnosis and initial phases but higher in the late ($1,092 vs $952) and terminal phases ($1,752 vs. $392), primarily because of higher net costs for inpatient care (Figure 1, S.  Table 6). In the terminal phase, net 30-day costs were also higher for those born before 1945 than for those born during 1945–1965 or after 1965 (-$1,617 vs. $406 vs. $1,261, respectively) (Figure 2, S.  Table 6).

### 3.4. Mean 1-Year, 5-Year, 10-Year, and 15-Year Net Healthcare Costs Adjusted for Survival

For Ontario immigrants, the mean net healthcare costs at 1 year, 5 years, 10 years, and 15 years were $4,307, $20,413, $44,398, and $90,448, respectively (Table 4). The mean net costs at 1, 5, and 10 years postindex were higher for males ($4,806, $22,707, and $47,650, respectively) than for females ($3,816, $18,154, and $41,514, respectively) but were slightly lower at 15 years after diagnosis ($90,693 for males and $92,042 for females). Mean net costs were also lower for those born before 1945. The same findings were held for undiscounted costs (Table 4).

## 4. Discussion

We conducted a population-level propensity-score-matched cohort study of incident cases of CHC among Ontario immigrants diagnosed between May 31, 2003, and December 31, 2018, to estimate CHC-attributable mortality, as well as phase-specific and long-term net healthcare costs. Our findings suggest that, compared to unexposed individuals, immigrants with CHC experience a significantly higher mortality rate and incur substantial healthcare costs that vary by phase of care and time horizon of analysis; the highest CHC-attributable costs were observed for the late phase. During the initial and late phases, outpatient prescriptions were the largest cost component, whereas, in the terminal phase, the costs were primarily driven by inpatient care. The average annual cost for immigrants with CHC was $6,120 during the initial phase and rose to $15,480 in the late phase, three times higher than Ontario's per capita healthcare spending of $5,400 in 2020 [19]. The substantial increase in costs highlights the role of early diagnosis and treatment in preventing CHC sequelae and reducing healthcare expenditure.

Our findings are generally consistent with those of previous HCV costing studies in Ontario, Canada. For instance, prior Ontario-based costing studies among the First Nations also found similar effects, with the largest burden of CHC-attributable healthcare costs in the late phase of care. The reported 30-day net costs (2018 CAD) incurred during the initial and terminal phases were consistent with our estimates but were found to be higher in the late phase of care ($1,768 ($1,814 in 2020 CAD) vs. $1,016) [8]. Another population-level costing study in Ontario by Wong et al. estimated CHC-related costs by disease stage but did not report net costs. They found the mean 30-day costs (2018 CAD) to vary by disease severity from $798 ($819 in 2020 CAD) for individuals with no cirrhosis to $1,487 ($1,526 in 2020 CAD) for those with compensated cirrhosis and to $3,659 ($3,755 in 2020 CAD) for decompensated cirrhosis [10]. The higher costs observed in advanced disease stages in those two studies could be attributed to a higher level of comorbidity and drug- or alcohol-related substance use disorder in the study populations compared to our study. These factors were found to be important predictors of CHC costs [10]. Although we did not evaluate the differences in costs between immigrants and non-immigrants in the current study, Wong et al. found significantly lower healthcare costs among immigrants experiencing non-advanced and advanced liver disease, but higher costs during the terminal care phase compared to non-immigrants [10].

In the present study, the 15-year net costs amounted to $102,881 CAD (undiscounted) and were higher for females and those born after 1965, possibly due to their longer life expectancy. Previous analyses have demonstrated an association between life expectancy and healthcare costs in the CHC population [20]. Notable, our long-term costs were ∼30% higher than the estimated lifetime costs of $64,694 ($70,654 in 2020 CAD) reported in a Canadian study of individuals with CHC in 2013 [21]. These differences may be attributed to variations in costing methods and the use of novel treatments. In Ontario, the cost of publicly funded antivirals is estimated to be over $50,000 per completed treatment course [22].

Our study has several limitations. First, given that the IRCC dataset starts in 1985, individuals identified as immigrants consist of only those who immigrated to Canada after 1985; thus, results may not be generalizable to earlier immigrants [23]. Second, although we were able to match a large proportion of HCV-exposed immigrants in Ontario, in general, the matched population tended to have lower levels of drug- or alcohol-related substance use disorder (1% vs. 36%) and lower comorbidity level (ADG >8 : 5.8% vs. 49%) compared to the unmatched population. Thus, our findings are less generalizable to immigrants with multiple comorbidities or a history of substance use disorders at the time of diagnosis. Indeed, in previous phase-based costing studies using similar methods among populations with a much higher proportion of substance use disorders, prediagnosis costs were found to be higher than in our study [8]. Third, we did not conduct stratified analysis by DAA era. However, it is important to note that the prior population-level costing study by Wong et al. demonstrated that outpatient drug costs constituted a substantial portion, ranging from 27% to 40% of total costs in the DAA era for individuals with fibrosis and compensated cirrhosis [10]. In contrast, our analysis revealed even higher percentages, with outpatient costs accounting for 65% and 48% of the total CHC-attributable costs during the initial (fibrosis, compensated cirrhosis) and late phases (decompensated cirrhosis, HCC) respectively. This notable difference suggests that a significant portion of treatment in our study may have occurred during the DAA era, given the high proportion of outpatient costs. Nonetheless, it is reasonable to anticipate that stratified analysis by DAA era would likely reveal an even greater proportion of outpatient costs in the DAA era. Lastly, as the current study is from the provincial healthcare payer perspective, the analysis does not include drug costs covered by private drug plans.

Our study has important strengths. Using population-level health administrative datasets and propensity-score-matching controls provided the first real-world estimate of CHC-attributable costs among immigrants in Ontario, Canada. We were also able to explore all important resource components, including inpatient and outpatient visits, emergency department visits, prescribed medications, laboratory and diagnostic services, and other services that account for CHC-attributable costs. Furthermore, using the phase-of-care approach, we estimated the cumulative long-term costs adjusted for survival.

The existing literature on costing in Ontario is limited, especially on diverse populations. To our knowledge, this is the first costing study to estimate CHC-attributable costs among immigrants in Ontario, Canada. Future studies should also report the results among different risk groups to gain a better understanding of the target population for whom interventions should be prioritized.

In conclusion, this study provides information on the economic burden of CHC among key risk groups in Ontario to help support provincial policymaking. Effective implementation of programs facilitating early diagnosis, treatment, and continuity of care among at-risk immigrant populations can significantly reduce the long-term health consequences and associated costs of untreated CHC, thereby alleviating the economic burden on the healthcare system. Our findings on CHC-attributable mortality and healthcare costs are important for planning and guiding economic evaluations of various HCV elimination efforts and public health interventions among Ontario immigrants living with hepatitis C.

## Figures and Tables

**Table 1:** Characteristics of the study cohort.

	Prematched			Matched			Unmatched
	Exposed *N* = 6,914	Unexposed *N* = 120,693	SMD	Exposed *N* = 5,575	Unexposed *N* = 5,575	SMD	Exposed *N* = 1,339
Index year^*∗*^, mean (sd)	2009 (4.0)	2013 (4.0)	0.74	2010 (4.0)	2010 (4.0)	0.00	2009 (4.5)
Age at diagnosis, years, mean (sd)	48.1 (16.1)	39.1 (17.7)	0.50	46.6 (14.8)	46.6 (14.8)	0.00	54.2 (19.6)
Birth year, *n* (%)							
<1945	1,141 (16.5)	7,799 (6.5)	0.32	663 (11.9)	663 (11.9)	0.00	478 (35.7)
1945–1965	2,895 (41.9)	29,993 (24.9)	0.37	2,474 (44.4)	2,474 (44.4)	0.00	421 (31.4)
>1965	2,878 (41.6)	82,901 (68.7)	0.57	2,438 (43.7)	2,438 (43.7)	0.00	440 (32.9)
Male sex, *n* (%)	3,648 (52.8)	58,413 (48.4)	0.09	2,891 (51.9)	2,891 (51.9)	0.00	757 (56.5)
Landing year, mean (sd)	2001 (8.0)	2003 (8.0)	0.26	2002 (8.0)	1999 (8.0)	0.38	1997 (7.5)
Years of education, mean (sd)	10.5 (5.5)	10.3 (6.3)	0.03	10.98 (5.3)	11.1 (5.1)	0.03	8.3 (5.9)
Rural, *n* (%)	64 (0.9)	1,333 (1.1)	0.02	54 (1.0)	46 (0.8)	0.02	10 (0.7)
Neighborhood income quintile, *n* (%)							
1st quintile (lowest)	2,490 (36.0)	36,752 (30.5)	0.12	1,909 (34.2)	1,946 (34.9)	0.01	581 (43.4)
2nd quintile	1,507 (21.8)	27,210 (22.5)	0.02	1,252 (22.5)	1,271 (22.8)	0.01	255 (19.0)
3rd quintile	1,286 (18.6)	23,042 (19.1)	0.01	1,071 (19.2)	1,074 (19.3)	0.00	215 (16.1)
4th quintile	1,054 (15.2)	19,581 (16.2)	0.03	880 (15.8)	797 (14.3)	0.04	174 (13.0)
5th quintile	577 (8.3)	14,108 (11.7)	0.11	463 (8.3)	487 (8.7)	0.02	114 (8.5)
Residential instability quintile, *n* (%)							
1st quintile (lowest)	1,817 (26.3)	32,216 (26.7)	0.01	1,528 (27.4)	1,522 (27.3)	0.00	289 (21.6)
2nd quintile	893 (12.9)	17,254 (14.3)	0.00	724 (13.0)	752 (13.5)	0.01	169 (12.6)
3rd quintile	790 (11.4)	14,934 (12.4)	0.03	659 (11.8)	645 (11.6)	0.01	131 (9.8)
4th quintile	1,128 (16.3)	19,091 (15.8)	0.01	882 (15.8)	956 (17.1)	0.04	246 (18.4)
5th quintile	2,286 (33.1)	37,198 (30.8)	0.05	1,782 (32.0)	1,700 (30.5)	0.03	504 (37.6)
Material deprivation quintile, *n* (%)							
1st quintile (lowest)	767 (11.1)	17,130 (14.2)	0.09	624 (11.2)	661 (11.9)	0.02	143 (10.7)
2nd quintile	888 (12.8)	18,514 (15.3)	0.07	745 (13.4)	685 (12.3)	0.03	143 (10.7)
3rd quintile	1,202 (17.4)	21,319 (17.7)	0.01	982 (17.6)	988 (17.7)	0.00	220 (16.4)
4th quintile	1,548 (22.4)	27,284 (22.6)	0.01	1,296 (23.2)	1,232 (22.1)	0.03	252 (18.8)
5th quintile	2,509 (36.3)	36,386 (30.1)	0.13	1,928 (34.6)	2,009 (36.0)	0.03	581 (43.4)
Ethnic concentration quintile, *n* (%)							
1st quintile (lowest)	145 (2.1)	2,759 (2.3)	0.01	103 (1.8)	113 (2.0)	0.01	42 (3.1)
2nd quintile	248 (3.6)	4,522 (3.7)	0.01	192 (3.4)	152 (2.7)	0.04	56 (4.2)
3rd quintile	491 (7.1)	9,443 (7.8)	0.03	372 (6.7)	371 (6.7)	0.00	119 (8.9)
4th quintile	1,366 (19.8)	24,479 (20.3)	0.01	1,082 (19.4)	981 (17.6)	0.05	284 (21.2)
5th quintile	4,664 (67.5)	79,490 (65.9)	0.03	3,826 (68.6)	3,958 (71.0)	0.05	838 (62.6)
Dependency quintile, *n* (%)							
1st quintile (lowest)	2,660 (38.5)	46,363 (38.4)	0.00	2,202 (39.5)	2,080 (37.3)	0.05	458 (34.2)
2nd quintile	1,695 (24.5)	28,288 (23.4)	0.03	1,384 (24.8)	1,322 (23.9)	0.02	311 (23.2)
3rd quintile	1,106 (16.0)	19,313 (16.0)	0.00	862 (15.5)	950 (17.0)	0.04	244 (18.2)
4th quintile	800 (11.6)	15,054 (12.5)	0.03	642 (11.5)	701 (12.6)	0.03	158 (11.8)
5th quintile	653 (9.4)	11,675 (9.7)	0.01	485 (8.7)	512 (9.2)	0.02	168 (12.5)
Substance use disorder, *n* (%)	496 (7.2)	1,321 (1.1)	0.31	59 (1.1)	59 (1.1)	0.00	437 (32.6)
HIV positivity, *n* (%)	27 (0.4)	21 (0.02)	0.08	20 (0.4)	<6 (<0.1)	0.07	7 (0.5)
ADG categories, *n* (%)							
0–3	3,489 (30.5)	84,704 (70.2)	0.41	3,267 (58.6)	3,267 (58.6)	0.00	222 (16.6)
4–7	2,445 (35.4)	29,971 (24.8)	0.23	1,984 (35.6)	1,984 (35.6)	0.00	461 (34.4)
8–10	668 (9.7)	4,946 (4.1)	0.22	289 (5.2)	289 (5.2)	0.00	379 (28.3)
>11	312 (4.5)	1,072 (0.9)	0.23	35 (0.6)	35 (0.6)	0.00	277 (20.7)

^*∗*^The index year is the year of CHC diagnosis. ADG: aggregated diagnostic groups; CHC: chronic hepatitis C; HIV: human immunodeficiency virus; *N*: number of observations; sd: standard deviation; SMD: standardized difference in means.

**Table 2:** Total and CHC-attributable mortality among immigrants by phase of care.

All-cause mortality	Exposed (*N* = 5,575)	Unexposed (*N* = 5,575)	CHC- attributable mortality (95% CI)
1-year (%)	1.33	0.20	ARR: 1.13 (0.81; 1.45)
			RR: 6.73 (3.57; 12.67)

5-year (%)	4.50	1.15	ARR: 3.35 (2.74; 3.97)
			RR: 3.92 (3.01; 5.12)

10-year (%)	8.43	2.57	ARR: 5.87 (5.03; 6.70)
			RR: 3.29 (2.76; 3.92)

15-year (%)	10.49	3.52	ARR: 6.98 (6.03; 7.92)
			RR: 2.98 (2.58; 3.46)

ARR: absolute risk reduction (%); CHC: chronic hepatitis C; RR: relative risk; CI: confidence interval.

**Table 3:** Total and CHC-attributable healthcare costs (in 2020 CAD) per 30 days among immigrants by phase of care.

Costs	Prediagnosis phase			Initial phase			Late phase			Terminal phase		
	Exposed	Unexposed	Net	Exposed	Unexposed	Net	Exposed	Unexposed	Net	Exposed	Unexposed	Net
	*N* = 5,575	*N* = 5,575	(95% CI)	*N* = 5,575	*N* = 5,575	(95% CI)	*N* = 1,535	*N* = 1,535	(95% CI)	*N* = 756	*N* = 756	(95% CI)
Total costs, mean (sd)	247 (953)	159 (848)	88 (55; 122)	510 (1,019)	187 (682)	324 (291; 356)	1,290 (2,054)	274 (864)	1,016 (901; 1,132)	8,967 (9,485)	7,992 (9,003)	975 (−25; 1,974)
Total costs, median (IQR)	83 (46–170)	32 (1–105)		196 (79–521)	61 (18–157)		565 (192–1,543)	78 (23–207)	—	6,109 (2,734–12,351)	5,760 (2,959–9,899)	—
Costs by resource category, mean (sd)												
Outpatient services	28 (350)	20 (270)	9 (−3; 20)	56 (378)	23 (192)	34 (22; 45)	171 (746)	42 (360)	129 (86; 172)	791 (1,604)	770 (1,610)	21 (−152; 195)
Physician services	83 (149)	57 (128)	26 (20; 31)	98 (138)	59 (81)	39 (34; 43)	202 (485)	73 (106)	129 (103; 155)	1,229 (1,134)	998 (932)	232 (119; 344)
ED visits	10 (36)	6 (27)	4 (3; 5)	9 (22)	6 (14)	3 (3; 4)	27 (88)	8 (23)	19 (14; 24)	229 (228)	180 (145)	49 (28; 69)
Same-day surgery	4 (33)	5 (40)	−1 (−2; 1)	9 (53)	6 (19)	3 (1; 4)	25 (46)	8 (26)	17 (14; 20)	35 (112.0)	36 (184)	−1 (−17; 16)
Inpatient care	53 (511)	35 (492)	19 (0; 38)	65 (450)	41 (411)	24 (8; 41)	253 (963)	57 (359)	196 (142; 249)	5,156 (7,810)	3,994 (7,530)	1,162 (332; 1,991)
Outpatient prescription	21 (130)	17 (106)	5 (1; 9)	237 (545)	26 (146)	211 (196; 226)	533 (967)	42 (149)	491 (440; 542)	481 (1,821)	326 (688)	156 (7; 305)
Laboratory services	31 (27)	8 (15)	22 (21; 23)	14 (18)	7 (8)	7 (6; 8)	18 (23)	7 (8)	10 (9; 11)	22 (32)	18 (28)	4 (1; 7)
Other services	17 (394)	15 (316)	2 (−11; 15)	27 (240)	25 (273)	2 (−8; 11)	76 (463)	51 (528)	25 (−12; 61)	1,023 (2,012)	1,671 (2,807)	−648 (−912; −385)

Costs, standardized to 30 days, were collected cumulatively over each phase and rounded to the nearest dollar. CAD: Canadian dollar; CHC: chronic hepatitis C; CI: confidence interval; IQR: interquartile range; SD: standard deviation. *Note.* Negative attributable costs observed, for example, in the terminal phase for “other services,” often occur because exposed individuals are hospitalized and do not incur costs outside the hospital, but unexposed do.

**Table 4:** Mean net costs (in 2020 CAD) adjusted for survival, total and stratified by sex and birth cohort.

	Total	By sex		By birth cohort		
		Male	Female	1945	1945/1965	>1965
*Undiscounted costs, mean (95% CI)*						
1-year	$4,307 ($3,808; $4,795)	$4,806 ($4,087; $5,522)	$3,816 ($3,128; $4,492)	$4,010 ($1,915; $6,096)	$4,502 ($3,718; $5,274)	$4,387 ($3,774; $5,000)
5-year	$21,019 ($18,622; $23,363)	$23,371 ($19,883; $26,843)	$18,685 ($15,424; $21,891)	$19,024 ($9,318; $28,689)	$21,910 ($18,071; $25,693)	$21,781 ($18,767; $24,794)
10-year	$47,841 ($42,383; $53,216	$51,231 ($43,469; $58,912)	$44,869 ($37,137; $52,502)	$39,668 ($20,461; $58,804)	$49,805 ($41,546; $57,965)	$49,740 ($41,489; $57,991)
15-year	$102,881 ($91,112; $114,622)	$102,677 ($86,515; $118,489)	$105,262 ($87,592; $122,833)	$76,466 ($44,461; $108,401)	$108,575 ($92,641; $124,412)	$103,111 ($79,990; $126,232)

*Discounted (1.5%) costs, mean (95% CI)*						
5-year	$20,413 ($18,086; $22,690)	$22,700 ($19,311; $26,072)	$18,145 ($14,978; $21,258)	$18,482 ($9,051; $27,873)	$21,280 ($17,552; $24,954)	$21,149 ($18,223; 24,074)
10-year	$44,398 ($39,333; $49,383)	$47,650 ($40,438; $54,790)	$41,514 ($34,355; $48,579)	$36,973 ($19,003; $54,875)	$46,215 ($38,527; $53,811)	$46,176 ($38,594; $53,758)
15-year	$90,448 ($80,102; $100,760)	$90,693 ($76,452; $104,636)	$92,042 ($76,567; $107,422)	$67,781 ($39,095; $96,400)	$95,384 ($81,272; $109,403)	$90,826 ($70,803; $110,849)

CI: confidence interval.

## Data Availability

The dataset from this study is held securely in a coded form at the ICES. While legal data-sharing agreements between ICES and data providers (e.g., healthcare organizations and government) prohibit ICES from making the dataset publicly available, access may be granted to those who meet prespecified criteria for confidential access, available at https://www.ices.on.ca/DAS (e-mail: das@ices.on.ca). The full dataset creation plan and underlying analytic code are available from the authors upon request, understanding that computer programs may rely upon coding templates or macros that are unique to ICES and are, therefore, either inaccessible or may require modification.

## Supplementary Materials

Supplementary Materials Table S1. drug identification numbers used to identify HCV antiviral treatment. Table S2. diagnostic and procedure codes used to identify CHC diagnosis and comorbidities. Table S3. detailed description of diagnostic, death, and procedure-related codes. Table S4. characteristics of the study cohort in the late phase. Table S5. characteristics of the study cohort in the terminal phase. Table S6. total and CHC-attributable healthcare costs (2020 CAD) among immigrants by phase of care, stratified by sex and birth cohort.

## References

[1] World Health Organization (2016). *Combating Hepatitis B and C to Reach Elimination by 2030*.

[2] Wong W. W. L., Erman A., Feld J. J., Krahn M. (2017). Model-based projection of health and economic effects of screening for hepatitis C in Canada. *Canadian Medical Association Journal Open*.

[3] Greenaway C., Makarenko I., Tanveer F., Janjua N. Z. (2018). Addressing hepatitis C in the foreign-born population: a key to hepatitis C virus elimination in Canada. *Canadian Liver Journal*.

[4] Greenaway C., Azoulay L., Allard R. (2017). A population-based study of chronic hepatitis C in immigrants and non-immigrants in Quebec, Canada. *Bone Marrow Concentrate Infectious Diseases*.

[5] Chen W., Tomlinson G., Krahn M., Heathcote J. (2012). Immigrant patients with chronic hepatitis C and advanced fibrosis have a higher risk of hepatocellular carcinoma. *Journal of Viral Hepatitis*.

[6] Erman A., Wong W. W. L., Feld J. J., Grootendorst P., Krahn M. D. (2020). The health impact of delaying direct-acting antiviral treatment for chronic hepatitis C: a decision-analytic approach. *Liver International*.

[7] Erman A., Krahn M. D., Hansen T. (2019). Estimation of fibrosis progression rates for chronic hepatitis C: a systematic review and meta-analysis update. *British Medical Journal Open*.

[8] Mendlowitz A., Bremner K. E., Walker J. D. (2021). Health care costs associated with hepatitis C virus infection in First Nations populations in Ontario: a retrospective matched cohort study. *Canadian Medical Association Journal Open*.

[9] Krajden M., Kuo M., Zagorski B., Alvarez M., Yu A., Krahn M. (2010). Health care costs associated with hepatitis C: a longitudinal cohort study. *Canadian Journal of Gastroenterology*.

[10] Wong W. W. L., Haines A., Bremner K. E. (2021). Health care costs associated with chronic hepatitis C virus infection in Ontario, Canada: a retrospective cohort study. *Canadian Medical Association Journal Open*.

[11] Erman A., Everett K., Wong W. W. L. (2023). Engagement with the HCV care cascade among high-risk groups: a population-based study. *Hepatology Communications*.

[12] Johns Hopkins Medicine (2022). Johns Hopkins ACG® system version 13.0. https://www.hopkinsacg.org/.

[13] Nanwa N., Kwong J. C., Feld J. J., Fangyun Wu C., Sander B. (2022). The mean attributable health care costs associated with hepatitis B virus in Ontario, Canada: a matched cohort study. *Canadian Liver Journal*.

[14] de Oliveira C., Pataky R., Bremner K. E. (2016). Phase-specific and lifetime costs of cancer care in Ontario, Canada. *Bone Marrow Concentrate Cancer*.

[15] Austin P. C. (2011). Optimal caliper widths for propensity-score matching when estimating differences in means and differences in proportions in observational studies. *Pharmaceutical Statistics*.

[16] Wodchis W. P., Bushmeneva K., Nikitovic M., McKillop I. (2013). Guidelines on person-level costing using administrative databases in Ontario. *Working Paper Series*.

[17] Yabroff K. R., Lamont E. B., Mariotto A. (2008). Cost of care for elderly cancer patients in the United States. *Journal of the National Cancer Institute: Journal of the National Cancer Institute*.

[18] Canadian Agency for Drugs & Technologies in Health (2017). *Guidelines for the Economic Evaluation of Health Technologies*.

[19] Statistics Canada (2020). Expenses of government classified by function. https://www150.statcan.gc.ca/n1/daily-quotidien/211126/dq211126a-eng.htm.

[20] Razavi H., Elkhoury A. C., Elbasha E. (2013). Chronic hepatitis C virus (HCV) disease burden and cost in the United States. *Hepatology*.

[21] Myers R. P., Krajden M., Bilodeau M. (2014). Burden of disease and cost of chronic hepatitis C virus infection in Canada. *Canadian Journal of Gastroenterology and Hepatology*.

[22] Ontario Ministry of Health (2023). Ontario drug Benefit formulary/comparative drug index. https://www.health.gov.on.ca/en/pro/programs/drugs/odbf_eformulary.aspx.

[23] Yasseen A. S., Kwong J. C., Feld J. J. (2021). Viral hepatitis C cascade of care: a population-level comparison of immigrant and long-term residents. *Liver International*.

