# Supplementary material for: Hepatitis C Attributable Healthcare Costs and Mortality among Immigrants: A Population-Based Matched Cohort Study
Source: Can J Gastroenterol Hepatol. 2024 Feb 24;2024:5573068. doi: 10.1155/2024/5573068 (PMC10908570; doi:10.1155/2024/5573068)
Supplement: Supplementary Materials — Table S1. drug identification numbers used to identify HCV antiviral treatment. Table S2. diagnostic and procedure codes used to identify CHC diagnosis and comorbidities. Table S3. detailed description of diagnostic, death, and procedure-related codes. Table S4. characteristics of the study cohort in the late phase. Table S5. characteristics of the study cohort in the terminal phase. Table S6. total and CHC-attributable healthcare costs (2020 CAD) among immigrants by phase of care, stratified by sex and birth cohort. [file 5573068.f1.docx]

**Supplementary Materials**

[**Table S1. Drug identification numbers used to identify HCV antiviral treatment** 2](#_Toc140742523)

[**Table S2. Diagnostic and procedure codes used to identify CHC diagnosis and comorbidities** 3](#_Toc140742524)

[**Table S3. Detailed description of diagnostic, death and procedure-related codes used** 7](#_Toc140742525)

[**Table S4. Characteristics of the study cohort in the late phase** 11](#_Toc140742526)

[**Table S5. Characteristics of the study cohort in the terminal phase** 13](#_Toc140742527)

[**Table S6. Total and CHC-attributable healthcare costs (2020 CAD) among immigrants by phase of care, stratified by sex and birth cohort** 15](#_Toc140742528)

# **Table S1. Drug identification numbers used to identify HCV antiviral treatment**

DIN: Drug identification number; DAA: direct-acting antivirals; PEG-IFN: pegylated interferon; IFN: Interferon; RBV: ribavirin.

# **Table S2. Diagnostic and procedure codes used to identify CHC diagnosis and comorbidities**

| **Condition** | **Dataset** | **ICD-9** | **ICD-10** | **Procedure**  **Code** | **Intervention Code** | **OHIP Fee Code** | **OCR** | **Death Registry**  **Code** |
| --- | --- | --- | --- | --- | --- | --- | --- | --- |
| **Cirrhosis**^†^ | **DAD**  **NACRS** | 456.1  571.2  571.5 | I85.9  I98.2  K70.3  K74.6  K71.7 | - | - | - | - | - |
|  | **OHIP** | - | - | - | - | 571 | - | - |
| **Decompensated Cirrhosis** | **DAD**  **NACRS** | 456.0  456.2  572.2  572.3  572.4  782.4  789.5 | I85.0  I98.20  I98.3  I86.4  K76.6  K76.7  K72.1  K72.9  R17  R18 | CCP 1006  CCP 6691 | 1.NA.13.BA-FA  1.NA.13.BA-X7  1.NA.13.BA-BD 1.KQ.76GP-NR  1.OT.52.HA | - | - | - |
|  | **OHIP** | - | - | - | - | J057  Z591  571^†^ | - | - |
|  | **ORDG** | - | - | - | - | - | - | **The main cause of death**: 5715, 5712, 5722, 5723, 5724, 5728, 4560  **Other causes of death:** K721, K729, K703, K704, K717, K74, K746, K766, K767, I85X, I982X, I983, I864 |
| **Hepatocellular carcinoma** | **DAD**  **NACRS** | 155.0 | C22.9  C22.0  81703  81803 | - | - | - | - | - |
|  | **ORGD** | - | - | - | - | - | - | **The main cause of death:** 1550  **Other cause of Death:** 81703, 81803 |
|  | **OCR** | - | - | - | - | - | **Morpho-logy**  81703, 81723, 81733 81743, 81753, 81803  **Topology:** C220 |  |
| **Liver transplant** | **DAD**  **NACRS** | V42.7  996.82 | Z94.4  T86.40  T86.41  T86.42  T86.43  T86.49  T86.9 | 624x | 1OA85LAXXK  1OA85VCXXK  1OA85WLXXI  1OA85WLXXK | - | - | - |
|  | **OHIP** | - | - | - | - | S265  S266  S274  S294  S295 | - | - |

| **HIV** | **DAD**  **NACRS** | 042  043  044 | B20  B21  B22  B23  B24 | - | - | - | - | - |
| --- | --- | --- | --- | --- | --- | --- | --- | --- |
| **Substance use disorder (related to drugs and alcohol use)** | **DAD**  **NACRS** | V113  V6542  291  292  303  304  3050  3052  3053  3054  3055  3056  357  3057  3058  3059  4255  5353  5710  5711  5712  5713  5771  6483  7903  980  965  967  968  969  E8500  E8501  E8502  E860  E9351 | 1426  F10  F11  F12  F13  F14  F16  F18  F19  G621  R780  R781  R782  R783  R784  R785  K70  K292  K860  T40  X65  Y91  Y573  Y919  Z502  Z503  Z714  Z715  Z721  Z864 | - | - | 303  304 | - | - |
|  | **OMHRS** | **DSM-4/5:** 291, 2920 2929, 303, 3040, 3041, 3042, 30423, 3044, 3045, 3046, 3048, 3050, 3052, 3053, 3054, 3055, 3056, 3057, 3059, 29211, 29212, 29281, 29283, 29284, 29289 | | | | | | |

†Cirrhosis was defined as a single inpatient cirrhosis code listed above. Decompensated cirrhosis was defined as cirrhosis using the outpatient cirrhosis code (OHIP: 571) and at least one inpatient diagnostic code, procedure code or death code associated with decompensated cirrhosis. HCC was defined as either a diagnosis in the Ontario Cancer Registry or inpatient diagnostic code or death code associated with hepatocellular carcinoma as listed above. The DAD dataset contains diagnostic and procedural data on inpatient hospital admissions. The NACRS dataset contains diagnostic and procedural information from ambulatory care and emergency department visits. The OHIP dataset contains data on claims made by physicians regarding universally insured services. OCR is the Ontario Cancer Registry and holds information on all Ontario residents with a cancer diagnosis, including the diagnosis date and details on the type of cancer. The ORGD dataset contains data on the date and cause of death of residents in Ontario. The OMHR dataset contains information on individuals receiving adult mental health services in Ontario and admissions to mental health–designated hospital beds.

CHC: chronic hepatitis C; DAD: Discharge Abstract Database; DSM: Diagnostic and Statistical Manual of Mental Disorders; HIV: human immunodeficiency virus; ICD-9: International Classification of Diseases, 9^th^ Revision, ICD-10: International Classification of Diseases, 10^th^ revision; NACRS: National Ambulatory Care Reporting System; OCR: Ontario Cancer Registry; OHIP: Ontario Health Insurance Program, OMHRS: Ontario Mental Health Reporting System; ORGD: Office of the Registrar General- Deaths.

# **Table S3. Detailed description of diagnostic, death and procedure-related codes used**

| **CIRRHOSIS** |  |  |
| --- | --- | --- |
|  | **ICD-9 Code** | **ICD-10 Code** |
| Toxic liver disease with fibrosis and cirrhosis of the liver |  | K.71.7 |
| Alcoholic cirrhosis | 571.2 | K.70.3 |
| Esophageal varices without bleeding | 456.1 | I.85.9, I.98.2 |
| Cirrhosis of the liver w/o alcohol | 571.5 | K.74.6 |
| **DECOMPENSATED CIRRHOSIS** |  |  |
|  | **ICD-9 Code** | **ICD-10 Code** |
| Portal hypertension | 572.3 | K.76.6 |
| Hepatorenal syndrome | 572.4 | K.76.7 |
| Jaundice | 782.4 | R.17 |
| Hepatic coma | 572.2 | - |
| Hepatic failure | - | K.72.1, K.72.9 |
| Esophageal varices with bleeding | 456.0, 456.2 | I.85.0, I.98.20, I.98.3 |
| Gastric varices | - | I.86.4 |
| Ascites | 789.5 | R.18 |
|  | **OHIP Code** | |
| Cirrhosis | 571^†^ | |
| Transjugular intrahepatic portosystemic shunt | J057 | |
| Paracentesis | Z591 | |
|  | **Intervention Code (CCI)** | **Procedure Code (CCP)** |
| Endoscopy for upper GI bleed | 1NA1BA-FA, 1NA13BA-X7, 1NA13BA-BD | - |
| Insertion of Sengstaken tube | - | 1006 |
| Transjugular intrahepatic portosystemic shunt | 1KQ76.GP-NR | - |
| Paracentesis | 1OT52.HA | 6691 |
| Decompensated cirrhosis | **The main cause of death** | **Other causes of death** |
|  | 5715 (Cirrhosis NOS)  5712 (Alcohol-related cirrhosis)  5722 (Hepatic coma)  5723 (Portal hypertension)  5724 (Hepatorenal sx)  5728 (Other sequelae chronic liver disease)  4560 (Esophageal varices with bleed) | K.72.1 (Chronic hepatic failure) K.72.9 (Hepatic failure, unspecified) K.70.3 (Alcoholic cirrhosis of the liver) K.70.4 (Alcoholic hepatic failure) K.71.7 (Toxic liver disease with fibrosis and cirrhosis of the liver) K.74 (Fibrosis and cirrhosis of the liver) K.74.6 (Other and unspecified cirrhosis of liver) K.76.6 (Portal hypertension) K.76.7 (Hepatorenal syndrome) I.85.X, I982X, I983 (Oesophageal varices) I864 (Gastric varices) |
| **HEPATOCELLULAR CARCINOMA** |  |  |
|  | **ICD-9 Code** | **ICD-10 Code** |
| Malignant neoplasm of liver | 155.0 | C.22.9 |
| Hepatocellular carcinoma | - | C.22.0, 81703 |
| Combined hepatocellular and cholangiocarcinoma | - | 81803 |
|  | **OCR Code (Morphology)** | **OCR Code (Topography)** |
| NOS | 81703 | C220 |
| Scirrhous | 81723 |  |
| Spindle | 81733 |  |
| Clear cell | 81743 |  |
| Pleomorphic | 81753 |  |
| Combined hepatocellular carcinoma (HCC) and cholangiocarcinoma | 81803 |  |
| Hepatocellular carcinoma | **The main cause of death** | **Other causes of death** |
|  | 1550 (Malignant Neoplasm of the Liver, Primary) | 81703 (HCC NOS)  81803 (Combined HCC and cholangiocarcinoma) |
| **HIV** |  |  |
|  | **ICD-9 Code** | **ICD-10 Code** |
| Human immunodeficiency virus [HIV] disease | 042, 0.43, 0.44 | B.20, B.21, B.22, B.23, B.24 |

| **LIVER TRANSPLANT** |  |  |
| --- | --- | --- |
|  | **ICD-9 Code** | **ICD-10 Code** |
| Liver replaced by transplant | V.42.7 | Z.94.4 |
| Complications of the transplanted liver | 996.82 | T.86.40, T.86.41, T.86.42, T.86.43, T.86.49, T.86.9 |
|  | **OHIP Code** | |
| Living donor, hepatectomy | S265 | |
| Living donor orthotopic liver transplantation recipient | S266 | |
| Donor, liver removal | S274 | |
| Liver excision, liver transplant recipient | S294 | |
| Digestive system-liver, repeat liver transplant | S295 | |
|  | **Intervention Code (CCI)** | |
| Transplant, liver of a deceased donor full-size liver | 1OA85LAXXK | |
| Transplant, liver of a deceased donor, multiorgan liver with intestine, pancreas, spleen, or stomach, or any combination of | 1OA85VCXXK | |
| Transplant, liver of a living donor, split liver | 1OA85WLXXJ | |
| Transplant, liver of a deceased donor split liver, or reduced pediatric-size liver | 1OA85WLXXK | |
| **SUBSTANCE USE** |  |  |
|  | **ICD-9 Code** | **ICD-10 Code** |
| Personal history of alcoholism | V.11.3 | Z.72.1 |
| Counseling on substance use and abuse | V.65.42 | Z.50.2, Z.50.3, Z.71.4, Z.71.5, Y.57.3 |
| Alcohol-induced mental disorders | 291 | F10 |
| Drug-induced mental disorders | 292 | F11, F12, F13, F14, F16, F18, F19 |
| Alcohol-dependence syndrome | 303 |  |
| Drug-dependence | 304 | Z.86.4 |
| Abuse of alcohol cannabis, hallucinogen, sedative, opioid, cocaine, amphetamine or related acting sympathomimetic, antidepressant type, Other, mixed, or unspecified drug abuse | 305.0, 305.2, 305.3, 305.4, 305.5, 305.6, 305.7, 305.8, 305.9 |  |
| Alcoholic polyneuropathy | 357.5 | G.62.1 |
| Alcoholic cardiomyopathy | 425.5 | I.42.6 |
| Alcoholic gastritis | 535.3 | K.29.2 |
| Alcohol-induced chronic pancreatitis | 577.1 | K.86.0 |
| Alcoholic fatty liver, hepatitis, cirrhosis liver damage | 571.0, 571.1, 571.2, 571.3 | K.70 |
| Drug dependence in the mother, but complicating pregnancy, childbirth, or the puerperium | 648.3 |  |
| Excess blood-alcohol level and finding of opiate drugs, cocaine, hallucinogen, psychotropic other drugs of addictive potential. | 790.3 | R.78.0, R781, R782 R783, R784, R785 |
| Toxic effect of alcohol | 980 |  |
| Poisoning by analgesics, antipyretics, and antirheumatics, poisoning by sedatives and hypnotics, poisoning by other central nervous system depressants and anesthetics, poisoning by psychotropic agents | 965, 967, 968, 969 | T.40, X.65, Y.91 |
| Accidental poisoning - heroin, methadone, opiates | E8500, E8501, E8502 |  |
| Accidental poisoning - alcohol | E860 |  |
| Adverse effects of methadone | E9351 |  |
|  | **DSM4/ DSM5** | |
| Alcohol intoxication or withdrawal, delirium | 291 | |
| Substance, sedative, hypnotic, or anxiolytic withdrawal | 2920 | |
| Substance-related disorder NOS | 2929 | |
| Alcohol intoxication/ dependence | 303 | |
| Dependence – opioid, sedative, cocaine, cannabis, amphetamine, hallucinogen, and others | 3040, 3041, 3042, 3043, 3044, 3045, 3046, 3048 | |
| Substance abuse – opioid, sedative, cocaine, cannabis, amphetamine, hallucinogen, and others | 3050, 3052, 3053, 3054, 3055, 3056, 3057, 3059 | |
| Substance-induced psychotic disorder | 29211, 29212 | |
| Substance intoxication delirium | 29281 | |
| Substance-induced persisting amnestic disorder | 29283 | |

†Cirrhosis was defined as a single inpatient cirrhosis code listed above. Decompensated cirrhosis was defined as cirrhosis using the outpatient cirrhosis code (OHIP: 571) and at least one inpatient diagnostic code, procedure code or death code associated with decompensated cirrhosis.

DSM: Diagnostic and Statistical Manual of Mental Disorders; CCI: Canadian Classification of Interventions; CCP: Canadian Classification of Procedures; GI: gastrointestinal; HCC: hepatocellular carcinoma; HIV: human immunodeficiency virus; ICD-9: International Classification of Diseases, 9^th^ revision; ICD-10: International Classification of Diseases, 10^th^ revision; OCR: Ontario Cancer Registry; OHIP: Ontario Health Insurance Program

# **Table S4. Characteristics of the study cohort in the late phase**

|  | **Pre-Matched** | | |  | **Matched** |  | **Unmatched** |
| --- | --- | --- | --- | --- | --- | --- | --- |
|  | **Exposed N=1,582** | **Unexposed N=5,575** | **SMD** | **Exposed N=1,535** | **Unexposed N=1,535** | **SMD** | **Exposed N=47** |
| **Index year**^†^**, mean (sd)** | 2009 (4.0) | 2010 (4.0) | 0.13 | 2009 (4.0) | 2009 (4.0) | 0.00 | 2010 (3.9) |
| **Age at diagnosis, years, mean (sd)** | 52.4 (13.1) | 46.6 (14.8) | 0.41 | 52.3 (13.1) | 52.3 (13.1) | 0.00 | 52.5 (14.2) |
| **Birth year, n (%)** |  |  |  |  |  |  |  |
| <1945 | 281 (17.8) | 663 (11.9) | 0.17 | 271 (17.7) | 271 (17.7) | 0.00 | 10 (21.3) |
| 1945-1965 | 938 (59.3) | 2,474 (44.4) | 0.30 | 914 (59.5) | 914 (59.5) | 0.00 | 24 (51.1) |
| >1965 | 363 (22.9) | 2,438 (43.7) | 0.45 | 350 (22.8) | 350 (22.8) | 0.00 | 13 (27.7) |
| **Male sex, n (%)** | 858 (54.2) | 2,891 (51.9) | 0.05 | 829 (54.0) | 829 (54.0) | 0.00 | 29 (61.7) |
| **Landing year, mean (sd)** | 2001 (8.0) | 1999 (8.0) | 0.26 | 2001 (8.0) | 1998 (7.0) | 0.44 | 1999 (10.2) |
| **Years of education, mean (sd)** | 10.8 (5.3) | 11.1 (5.1) | 0.07 | 10.9 (5.2) | 11.2 (5.0) | 0.06 | 7.8 (5.5) |
| **Rural, n (%)** | 18 (1.1) | 46 (0.8) | 0.21 | 18 (1.2) | 17 (1.1) | 0.01 | 0 (0.0) |
| **Neighborhood income quintile, n (%)** | |  |  |  |  |  |  |
| 1^st^ quintile (lowest) | 534 (33.8) | 1,946 (34.9) | 0.02 | 520 (33.9) | 514 (33.5) | 0.01 | 14 (29.8) |
| 2^nd^ quintile | 347 (21.9) | 1,271 (22.8) | 0.02 | 340 (22.1) | 354 (23.1) | 0.02 | 7 (14.9) |
| 3^rd^ quintile | 297 (18.8) | 1,074 (19.3) | 0.01 | 289 (18.8) | 291 (19.0) | 0.00 | 8 (17.0) |
| 4^th^ quintile | 269 17.0) | 797 (14.3) | 0.07 | 258 (16.8) | 228 (14.9) | 0.05 | 11 (23.4) |
| 5^th^ quintile | 135 (8.5) | 487 (8.7) | 0.01 | 128 (8.3) | 148 (9.6) | 0.05 | 7 (14.9) |
| **Residential instability quintile, n (%)** | |  |  |  |  |  |  |
| 1^st^ quintile (lowest) | 448 (28.3) | 1,522 (27.3) | 0.02 | 436 (28.4) | 409 (26.6) | 0.04 | 12 (25.5) |
| 2-3^rd^ quintiles | 403 (25.5) | 1,397 (25.1) | 0.01 | 393 (25.6) | 410 (26.7) | 0.04 | 10 (21.3) |
| 4^th^ quintile | 257 (16.2) | 956 (17.1) | 0.02 | 246 (16.0) | 256 (16.7) | 0.02 | 11 (23.4) |
| 5^th^ quintile | 474 (30.0) | 1,700 (30.5) | 0.01 | 460 (30.0) | 460 (30.0) | 0.00 | 14 (29.8) |
| **Material deprivation quintile, n (%)** | |  |  |  |  |  |  |
| 1-2^nd^ quintiles | 403 (25.5) | 1,346 (24.1) | 0.04 | 386 (25.1) | 401 (26.1) | 0.06 | 17 (36.2) |
| 3^rd^ quintile | 276 (17.4) | 988 (17.7) | 0.01 | 267 (17.4) | 282 (18.4) | 0.03 | 9 (19.1) |
| 4^th^ quintile | 383 (24.2) | 1,232 (22.1) | 0.05 | 377 (24.6) | 343 (22.3) | 0.05 | 6 (12.8) |
| 5^th^ quintile | 520 (32.9) | 2,009 (36.0) | 0.07 | 505 (32.9) | 509 (33.2) | 0.01 | 15 (31.9) |
| **Ethnnic concentration quintile, n (%)** | |  |  |  |  |  |  |
| 1-3^rd^ quintiles | 180 (11.4) | 636 (11.4) | 0.04 | 173 (11.3) | 204 (13.3) | 0.05 | 7 (14.9) |
| 4^th^ quintile | 305 (19.3) | 981 (17.6) | 0.04 | 285 (18.6) | 249 (16.2) | 0.06 | 20 (42.6) |
| 5^th^ quintile | 1,097 (69.3) | 3,958 (71.0) | 0.04 | 1,077 (70.2) | 1,082 (70.5) | 0.01 | 20 (42.6) |
| **Dependency quintile, n (%)** |  |  |  |  |  |  |  |
| 1^st^ quintile (lowest) | 617 (39.0) | 2,080 (37.3) | 0.03 | 592 (38.6) | 562 (36.6) | 0.04 | 25 (53.2) |
| 2^nd^ quintile | 418 (26.4) | 1,322 (23.9) | 0.06 | 408 (26.6) | 365 (23.8) | 0.06 | 10 (21.3) |
| 3-5^th^ quintiles | 547 (34.6) | 2,163 (38.8) | 0.06 | 535 (34.8) | 608 (39.6) | 0.05 | 12 (25.5) |
| **Substance use disorder, n (%)** | 14 (0.9) | 59 (1.1) | 0.02 | 9-14 (<0.9) | 13 (0.8) | 0.00 | <6 (<12.8) |
| **HIV positivity, n (%)** | 6 (0.4) | <6 (<0.1) | 0.08 | 6 (0.4) | 0 (0.0) | 0.09 | 0 (0.0) |
| **ADG categories, n (%)** |  |  |  |  |  |  |  |
| 0-3 | 853 (53.9) | 3,267 (58.6) | 0.09 | 830 (54.1) | 830 (54.1) | 0.00 | 23 (48.9) |
| ≥4 | 729 (46.1) | 2,308 (41.4) | 0.06 | 705 (45.9) | 705 (45.9) | 0.00 | 24 (51.1) |

†Index year is the year of late phase onset, that is, 3 months prior to advanced liver disease diagnosis.

ADG: aggregated diagnostic groups; HIV: human immunodeficiency virus; N: number of observations. sd: standard deviation; SMD: standardized difference in means

# **Table S5. Characteristics of the study cohort in the terminal phase**

|  | **Pre-Matched** | | | **Matched** | |  | **Unmatched** |
| --- | --- | --- | --- | --- | --- | --- | --- |
|  | **Exposed N=1,070** | **Unexposed N=2,329** | **SMD** | **Exposed N=756** | **Unexposed N=756** | **SMD** | **Exposed N=314** |
| **Index year**^†^**, mean (sd)** | 2015 (4.5) | 2017 (3.9) | 0.48 | 2016 (3.9) | 2016 (3.9) | 0.00 | 2012 (4.6) |
| **Age at death, years, mean (sd)** | 67.3 (16.0) | 72.1(16.9) | 0.29 | 67.7 (14.7) | 75.5 (13.2) | 0.56 | 66.2 (18.5) |
| **Birth year, n (%)** |  |  |  |  |  |  |  |
| <1945 | 524 (49.0) | 1,324 (56.8) | 0.16 | 353 (46.7) | 497 (65.7) | 0.39 | 171 (54.5) |
| 1945-1965 | 405 (37.9) | 740 (31.8) | 0.13 | 316 (41.8) | 229 (30.3) | 0.24 | 89 (28.3) |
| >1965 | 141 (13.2) | 265 (11.4) | 0.05 | 87 (11.5) | 30 (4.0) | 0.29 | 54 (17.2) |
| **Male sex, n (%)** | 608 (56.8) | 1,221 (52.4) | 0.09 | 426 (56.3) | 426 (56.3) | 0.00 | 182 (58.0) |
| **Landing year, mean (sd)** | 1998 (8.0) | 1998 (8.0) | 0.04 | 1999 (8.0) | 1998 (7.9) | 0.07 | 1997 (7.5) |
| **Years of education, mean (sd)** | 9.6 (5.4) | 9.9(5.4) | 0.05 | 9.8 (5.4) | 9.7 (5.4) | 0.02 | 9.0 (5.4) |
| **Rural, n (%)** | 12 (1.1) | 32 (1.4) | 0.02 | <6 (<0.8) | 11 (1.5) | 0.01 | <6 (<1.9) |
| **Neighborhood income quintile, n (%)** | |  |  |  |  |  |  |
| 1^st^ quintile (lowest) | 414 (38.7) | 752 (32.3) | 0.13 | 271 (35.8) | 264 (34.9) | 0.02 | 143 (45.5) |
| 2^nd^ quintile | 220 (20.6) | 519 (22.3) | 0.04 | 158 (20.9) | 158 (20.9) | 0.00 | 62 (19.7) |
| 3^rd^ quintile | 173 (16.2) | 417 (17.9) | 0.05 | 130 (17.2) | 118 (15.6) | 0.04 | 43 (13.7) |
| 4^th^ quintile | 161 (15.0) | 394 (16.9) | 0.05 | 116 (15.3) | 122 (16.1) | 0.02 | 45 (14.3) |
| 5^th^ quintile | 102 (9.5) | 247 (10.6) | 0.04 | 81 (10.7) | 94 (12.4) | 0.05 | 21 (6.7) |
| **Residential instability quintile, n (%)** | |  |  |  |  |  |  |
| 1^st^ quintile (lowest) | 289 (27.0) | 692 (29.7) | 0.06 | 228 (30.2) | 227 (30.0) | 0.00 | 61 (19.4) |
| 2^nd^ quintile | 124 (11.6) | 343 (14.7) | 0.09 | 99 (13.1) | 86 (11.4) | 0.05 | 25 (8.0) |
| 3^rd^ quintile | 132 (12.3) | 239 (10.3) | 0.07 | 88 (11.6) | 88 (11.6) | 0.00 | 44 (14.0) |
| 4^th^ quintile | 183 (17.1) | 373 (16.0) | 0.03 | 114 (15.1) | 128 (16.9) | 0.05 | 69 (22.0) |
| 5^th^ quintile | 342 (32.0) | 682 (29.3) | 0.06 | 227 (30.0) | 227 (30.0) | 0.00 | 115 (36.6) |
| **Material deprivation quintile, n (%)** | |  |  |  |  |  |  |
| 1^st^ quintile (lowest) | 123 (11.5) | 273 (11.7) | 0.01 | 94 (12.4) | 90 (11.9) | 0.02 | 29 (9.2) |
| 2^nd^ quintile | 137 (12.8) | 363 (15.6) | 0.08 | 104 (13.8) | 116 (15.3) | 0.05 | 33 (10.5) |
| 3^rd^ quintile | 165 (15.4) | 433 (18.6) | 0.08 | 120 (15.9) | 115 (15.2) | 0.02 | 45 (14.3) |
| 4^th^ quintile | 245 (22.9) | 534 (22.9) | 0.00 | 176 (23.3) | 180 (23.8) | 0.01 | 69 (22.0) |
| 5^th^ quintile | 400 (37.4) | 726 (31.2) | 0.13 | 262 (34.7) | 255 (33.7) | 0.02 | 138 (43.9) |
| **Ethnic concentration quintile, n (%)** | |  |  |  |  |  |  |
| 1^st^ quintile (lowest) | 25 (2.3) | 56 (2.4) | 0.00 | 17 (2.2) | 12 (1.6) | 0.05 | 8 (2.5) |
| 2^nd^ quintile | 38 (3.6) | 101 (4.3) | 0.04 | 28 (3.7) | 25 (3.3) | 0.02 | 10 (3.2) |
| 3^rd^ quintile | 76 (7.1) | 205 (8.8) | 0.06 | 59 (7.8) | 66 (8.7) | 0.03 | 17 (5.4) |
| 4^th^ quintile | 231 (21.6) | 452 (19.4) | 0.05 | 160 (21.2) | 164 (21.7) | 0.01 | 71 (22.6) |
| 5^th^ quintile | 700 (65.4) | 1,515 (65.0) | 0.01 | 492 (65.1) | 489 (64.7) | 0.01 | 208 (66.2) |
| **Dependency quintile, n (%)** |  |  |  |  |  |  |  |
| 1^st^ quintile (lowest) | 359 (33.6) | 743 (31.9) | 0.04 | 252 (33.3) | 262 (34.7) | 0.03 | 107 (34.1) |
| 2^nd^ quintile | 254 (23.7) | 534 (22.9) | 0.02 | 187 (24.7) | 176 (23.3) | 0.03 | 67 (21.3) |
| 3^rd^ quintile | 172 (16.1) | 356 (15.3) | 0.02 | 106 (14.0) | 104 (13.8) | 0.01 | 66 (21.0) |
| 4^th^ quintile | 142 (13.3) | 326 (14.0) | 0.02 | 99 (13.1) | 108 (14.3) | 0.03 | 43 (13.7) |
| 5^th^ quintile | 143 (13.4) | 370 (15.9) | 0.07 | 112 (14.8) | 106 (14.0) | 0.02 | 31 (9.9) |
| **Substance use disorder, n (%)** | 119 (11.1) | 87 (3.7) | 0.28 | 40 (5.3) | 46 (6.1) | 0.03 | 79 (25.2) |
| **HIV positivity, n (%)** | <6 (<0.6) | <6 (<0.3) | 0.06 | <6 (<0.8) | <6 (<0.8) | 0.00 | <6 (<1.9) |
| **ADG categories, n (%)** |  |  |  |  |  |  |  |
| 0-3 | 325 (30.4) | 1,013 (43.5) | 0.27 | 277 (36.6) | 220 (29.1) | 0.16 | 48 (15.3) |
| 4-7 | 422 (39.4) | 887 (38.1) | 0.03 | 328 (43.4) | 328 (43.4) | 0.00 | 94 (29.9) |
| 8-10 | 190 (17.8) | 295 (12.7) | 0.14 | 105 (13.9) | 135 (17.9) | 0.11 | 85 (27.1) |
| >11 | 133 (12.4) | 134 (5.8) | 0.23 | 46 (6.1) | 73 (9.7) | 0.13 | 87 (27.7) |

†Index year is the year of death.

ADG: aggregated diagnostic groups; HIV: human immunodeficiency virus; N: number of observations. sd: standard deviation; SMD: standardized difference in means

# **Table S6. Total and CHC-attributable healthcare costs (2020 CAD) among immigrants by phase of care, stratified by sex and birth cohort**

| **Cost,**  **mean (sd)** | **N** | **Exposed** | **Unexposed** | **Net (95% CI)** | |
| --- | --- | --- | --- | --- | --- |
|  |  |  | **Pre-diagnosis phase** | |  |
| Men | 2,891 | 232 (918) | 126 (791) | | 105 (61; 150) |
| Women | 2,684 | 263 (989) | 194 (905) | | 69 (19; 120) |
| <1945 | 663 | 557 (1,974) | 402 (1,777) | | 155 (-47; 358) |
| 1945-1965 | 2,474 | 228 (841) | 159 (822) | | 69 (23; 115) |
| >1965 | 2,438 | 181 (520) | 92 (285) | | 89 (65; 113) |
|  |  |  | **Initial phase** | |  |
| Men | 2,891 | 520 (1,186) | 153 (553) | | 367 (319; 415) |
| Women | 2,684 | 500 (804) | 224 (796) | | 277 (233; 320) |
| <1945 | 663 | 645 (1,201) | 471 (972) | | 174 (52; 295) |
| 1945-1965 | 2,474 | 529 (973) | 197 (797) | | 332 (281; 382) |
| >1965 | 2,438 | 460 (1,011) | 99 (364) | | 361 (318; 404) |
|  |  |  | **Late phase** | |  |
| Men | 829 | 1,211 (1,955) | 261 (842) | | 952 (799; 1100) |
| Women | 706 | 1,381 (2,161) | 289 (889) | | 1,092 (914; 1270) |
| <1945 | 271 | 1,598 (2,058) | 666 (1,467) | | 932 (616; 1248) |
| 1945-1965 | 914 | 1,307 (1,943) | 223 (705) | | 1,084 (945; 1223) |
| >1965 | 350 | 1,021 (2,289) | 109 (434) | | 912 (661; 1163) |
|  |  |  | **Terminal phase** | |  |
| Men | 426 | 8,606 (9,642) | 8,215 (7,905) | | 392 (-869; 1652) |
| Women | 330 | 9,443 (9,269) | 7,691 (10,312) | | 1,752 (131; 3373) |
| <1945 | 353 | 8,586 (8,634) | 7,325 (7,151) | | 1,261 (144; 2379) |
| 1945-1965 | 316 | 9,888 (10,465) | 9,482 (12,349) | | 406 (-1722; 2534) |
| >1965 | 87 | 7,355 (9,183) | 8,972 (8,453) | | -1,617 (-5,820; 2,586) |

CAD: Canadian dollar; CI: confidence interval; CHC: chronic hepatitis C; sd: standard deviation.

Note: Negative attributable costs observed, for example, in the terminal phase for “other services”, often occur because exposed individuals are hospitalized and do not incur costs outside the hospital, but unexposed do.
